# Spatial and Temporal Gait Characteristics in Patients Admitted to a Neuro-Rehabilitation Department with Age-Related White Matter Changes: A Gait Analysis and Clinical Study

**DOI:** 10.3390/neurolint15020044

**Published:** 2023-05-25

**Authors:** Andrea Gagliardo, Antonello Grippo, Vincenzo Di Stefano, Riccardo Carrai, Maenia Scarpino, Monica Martini, Catiuscia Falsini, Giulia Rimmaudo, Filippo Brighina

**Affiliations:** 1 IRCCS Fondazione Don Carlo Gnocchi, 50143 Firenze, Italy; antonello.grippo@unifi.it (A.G.); riccardo.carrai@unifi.it (R.C.); scarpinom@aou-careggi.toscana.it (M.S.); mmartini@dongnocchi.it (M.M.); cfalsini@dongnocchi.it (C.F.); 2 Clinical Neurophysiology Unit, “Clinical Course”, 90143 Palermo, Italy; giulia.rimmaudo2000@gmail.com; 3 Department of Biomedicine, Neuroscience and Advanced Diagnostic, University of Palermo, 90127 Palermo, Italy; vincenzo19689@gmail.com (V.D.S.); filippo.brighina@gmail.com (F.B.); 4 SODc Neurofisiopatologia, Dipartimento Neuromuscoloscheletrico e degli Organi di Senso, AOU Careggi, 50134 Firenze, Italy

**Keywords:** gait analysis, geriatric, age-related white matter changes, depression, gait disorders

## Abstract

Background: Patients with age-related white matter changes (ARWMC) frequently present a gait disorder, depression and cognitive impairment. Our aims are to define which alterations in the gait parameters are associated with motor or neuro-psychological impairment and to assess the role of motor, mood or cognitive dysfunction in explaining the variance of the gait parameters. Methods: Patients with gait disorders admitted to a Neuro-rehabilitation Department, affected by vascular leukoencephalopathy who had ARWMC confirmed by a brain MRI, were consecutively enrolled, classified by a neuroradiological scale (Fazekas 1987) and compared to healthy controls. We excluded subjects unable to walk independently, subjects with hydrocephalus or severe aphasia, with orthopaedic and other neurological pathologies conditioning the walking pattern. Patients and controls were assessed by clinical and functional scales (Mini Mental State Examination, Geriatric Depression Scale, Nevitt Motor Performance Scale, Berg Balance Scale, Functional Independence Measure), and computerised gait analysis was performed to assess the spatial and temporal gait parameters in a cross-sectional study. Results: We recruited 76 patients (48 males, aged 78.3 ± 6.2 years) and 14 controls (6 males, aged 75.8 ± 5 years). In the multiple regression analysis, the gait parameter with overall best model summary values, associated with the ARWMC severity, was the stride length even after correction for age, sex, weight and height (R^2^ = 0.327). The motor performances justified at least in part of the gait disorder (R^2^ change = 0.220), but the mood state accounted independently for gait alterations (R^2^ change = 0.039). The increase in ARWMC severity, the reduction of motor performance and a depressed mood state were associated with a reduction of stride length (R = 0.766, R^2^ = 0.587), reduction of gait speed (R^2^ = 0.573) and an increase in double support time (R^2^ = 0.421). Conclusion: The gait disorders in patients with ARWMC are related to motor impairment, but the presence of depression is an independent factor for determining gait alterations and functional status. These data pave the way for longitudinal studies, including gait parameters, to quantitatively assess gait changes after treatment or to monitor the natural progression of the gait disorders.

## 1. Introduction

Gait disorders are commonly found in old people, and their prevalence builds up with increasing age; at the age of 60, 85% of people have gait parameters that are comparable to the mean elderly population, but at the age of 85 or older, this proportion drops to 18% [1,2]. The prevalence of balance difficulties among older adults is estimated to be up to 20% [3,4,5]. The cumulative and extreme effects of normal ageing, superimposed by the negative effects of inactivity and multiple chronic diseases, represent geriatric frailty.

In patients with chronic vascular leukoencephalopathy, the white matter abnormalities have been associated with neurological, neuropsychological and behavioural disorders [6]. Since age represents the strongest factor associated with these alterations, the term age-related white matter changes (ARWMC) have been proposed [7]. Neuroimaging has also promoted the “vascular’’ theory to explain gait changes in relation to cognitive disorders linked with ageing [8]; the subcortical vascular lesions are a significant predictor for the risk of falls in elderly populations [6,7] and this could be related to the subcortical hypoperfusion interrupting connections critical for gait and balance and mediated by deep white matter sensory and motor tracts [9,10].

Studies on humans and animals actually support the hypothesis that cognitive decline associated with ageing [11] actually depends on the disturbances in white matter (WM), including myelin damage; thus, age-related changes in WM may contribute to the functional decline observed in the elderly [12].

In subjects with ARWMC, gait alteration has an onset with an ataxic-dyspraxic aspect and is associated with the imbalance and risk of falls [13,14]. These disturbances have a progressive course and are characterised by a short-stepped, widened base gait, with start and turnaround hesitation, a tendency to retropulsion and trunk and limb rigidity. Gait alterations are a predictor of falling in the elderly [15]; gait speed is an important measure of functional ability and has been widely used in older adults as an indicator of frailty, too [16].

### Aims of the Study

Few studies have quantitatively analysed both gait alteration and clinical-radiological findings in subjects with ARWMC [17,18,19] and the apparent relationship between the progression of gait abnormalities and the amount of leukoencephalopathy; however, no one has still examined if gait alterations related to ARWMC are associated with motor, mood or cognitive disorders.

We conducted a cross-sectional study in a sample of patients with ARWMC from our Neurorehabilitation department. We studied clinical and functional outcomes through apposite measurable scales and computerised gait data with the aims to: 

(a) Evaluate changes of gait parameters according to the severity of ARWMC; 

(b) Define which gait parameters alteration is associated with the motor and neuro-psychological impairment; 

(c) To assess the role of motor, mood or cognitive dysfunctions in explaining the variance of the gait parameters. 

## 2. Materials and Methods

### 2.1. Patients and Controls

Patients with a gait disorder, admitted to the Neuro-rehabilitation Department of the Don Carlo Gnocchi Foundation in Florence and affected by vascular leukoencephalopathy, were consecutively enrolled over a period of 24 months, from January 2008 to December 2009. We excluded subjects unable to walk independently, subjects with hydrocephalus or severe aphasia, with orthopaedic and other neurological pathologies conditioning the walking pattern. Furthermore, we recruited a sex- and age-matched control group, chosen among our patients’ relatives, who had no MRI-detectable alterations in the brain. The study was approved by the Don Gnocchi Foundation’s internal ethical board, and informed consent was obtained from all participants before involvement in the study.

### 2.2. Neuroimaging Data

Patients had ARWMC confirmed by a previous neuroimaging; a second brain scan with the same field strength (1.5 T) and comparable sequences [20] was performed in each patient within three months from inclusion in the study. A trained Neurologist (A.G.) classified the cerebral white matter changes into three categories (mild, moderate and severe ARWMC), according to the modified Fazekas visual scale [20].

### 2.3. Clinical and Functional Assessments

Clinical Evaluation: an overall cognitive evaluation was performed using the Mini Mental State Examination (MMSE) [21] corrected for age and years of education, in order to obtain a short 30-item score; mood state was assessed by the Geriatric Depression Scale questionnaire (GDS), a 30 item score with a yes/no format which best fitted for a geriatric population [22]; functional level was obtained by the Functional Independence Measure (FIM) scale [23] that can detect changes in dependence occurring in a rehabilitation department; motor performance during walking and postural stability by the Nevitt Motor Performance (NMPS) [24] as a clinical scale for gait and the Berg Balance Scales (BBS) [25,26] to objectively determine a patient’s ability (or inability) to safely balance during a series of predetermined tasks.

### 2.4. Gait Analysis

In order to analyse the spatial-temporal parameters of gait, we recorded each subject during walking using a computerised Gait Analysis system (SMART System v1.10 BTS S.p.a., Milan, Italy) consisting of six infrared camcorders placed around the edge of a lab of 9 × 7 m.

The positioning of the retro-reflective anatomically aligned markers on the lower limbs was performed according to the Davis Protocol [27]. The patients were recorded during a barefoot walk on a 7-m-long walkway at a self-selected speed. Two strides at the beginning and at the end of each walk were excluded from the succeeding analysis; four walking trials were analysed and the following gait parameters were averaged: stance, swing and double support times (%), stride time (s), cadence (step/min), step length and width (m), swing speed (m/s), walking speed (m/s) and stride length (m). To reduce the variability of gait parameters due to anthropometric measures, the temporal parameters were normalised in the percentage of the walking cycle and the spatial parameters in the percentage of patients’ height. 

### 2.5. Statistical Analysis

We calculated the averages and standard deviations of both gait parameters and clinical scores of patients grouped according to ARWMC severity. The association of ARWMC severity (mild, moderate and severe: categorical independent variables) with both gait parameters and clinical-functional scores (dependent variables) was tested using a univariate analysis of variance (ANOVA); for an inter-group comparison of patients with different ARWMC severity and controls, we performed a post hoc Least Significant Difference (LSD) analysis. 

In order to analyse the association of clinical scores (cognition, mood, functional level, motor performance and balance: ordinal independent variables) with gait parameters (continuous independent variables) and their univariate linear relationship, we calculated the Pearson Correlation Coefficients. 

The association of ARWMC severity with gait data was first explored by a linear regression model corrected for age, sex, weight and height. 

In addition, the adjusted ARWMC severity, cognition, mood, functional level, motor performance and balance scores were entered as independent variables into a multiple linear regression model. Their effect on each gait parameter (dependent variables) was examined with a “forward stepwise” selection method. The reason for which we performed a forward stepwise method was to select the best-predicting variable for entry in the model at each step and remove the insignificant variables at once until no more significant variables were present. The first variable considered for entry into the equation was the one with the largest positive or negative correlation with the dependent variable. This variable was entered into the equation only if it satisfied the criterion for entry (*p* < 0.05). If the first variable was entered, the independent variable not in the equation that had the largest partial correlation with the dependent variable was considered next. The procedure stopped when there were no variables that met the entry criterion. To understand how well the regression variables in the model truly represented our set of data, we analysed the multiple correlation coefficient value (R), which is the linear correlation between the observed and the model-predicted values of the dependent variable and the coefficient of determination values (R2), which represent the proportion of variation explained by each regression model, and their changes at each step. Best Coefficient Values (R and R2) are ideally considered proximal to 1.0. A correlation (R) greater than 0.8 is generally described as strong, whereas a correlation less than 0.5 is generally described as weak. For statistics, values of *p* < 0.05 were considered significant. Statistical computations were performed by SPSS 16.0.2 (SPSS Inc., Chicago, IL, USA) program.

## 3. Results

### 3.1. Sociodemographic Data

We recruited 76 patients (48 males, mean age 78.3 ± 6.2 years) with ARWMC and 14 controls (6 males, 75.8 ± 5 years); 47.3% of the patients had severe, 32.9% moderate and 19.7% mild ARWMC, according to the Fazekas classification (1987). The patients’ and controls’ demographic and anthropometric data are reported in Table 1. The control group did not show significant differences in age, sex and height compared with the three groups of patients (post hoc LSD *p* > 0.05). We did not find a significant difference between left and right gait parameters (*t*-Test *p* > 0.05) in either patients or controls, so for further analysis we used the mean value for each subject.

### 3.2. Clinical and Functional Data

The MMSE score was lower in the moderate subjects if compared to the control subjects and was as well lower in the severe than in both mild and moderate ARWMC. The patients’ group had a mean depression score higher than the controls; the mean GDS values in the three ARWMC groups did not differ significantly. The functional level (FIM) was significantly lower in the moderate group vs controls and the severe vs moderate ARWMC. Motor performance (NMPS) and balance (BBS) were lower in the mild vs. controls, and in the moderate vs. mild ARWMC. We found no difference in motor performance and balance between severe and moderate ARWMC. The functional decline (FIM) shown in the severe versus moderate group was significantly different only in the cognitive scores (*p*< 0.05 post hoc LSD, Table 1). 

### 3.3. Gait Analysis Data

**Figure 1:**
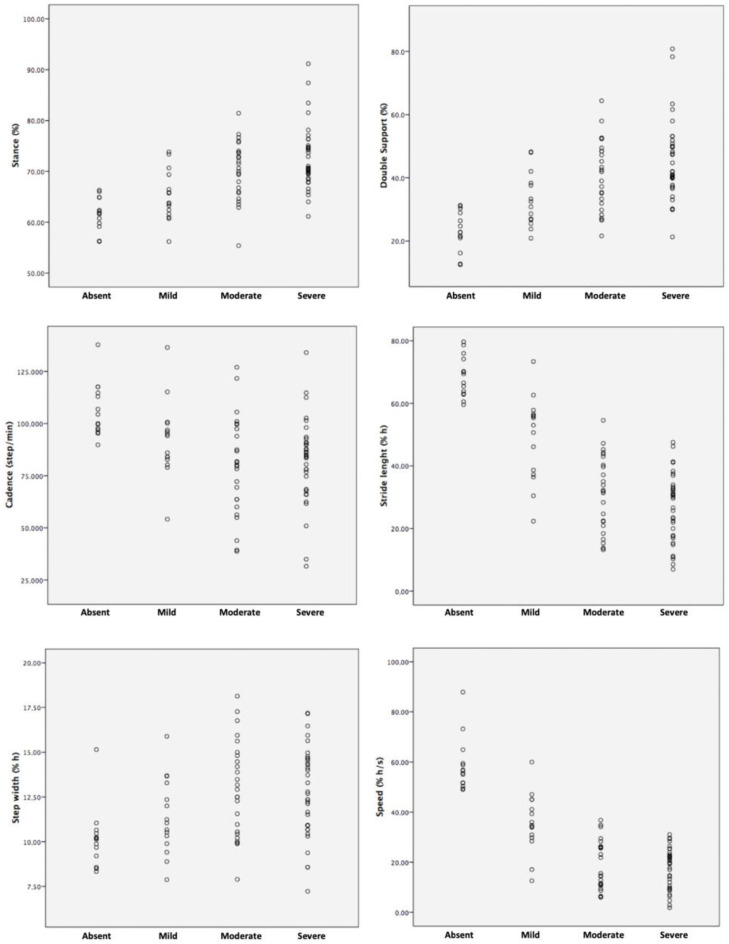
Scatterplots of gait parameters in subjects with different ARWMC severity. Abbreviations: ARWMC = Age-related white matter changes.

The mean ± standard deviation (SD), ANOVA and post hoc LSD significance are reported in Table 1. In subjects with mild ARWMC, swing phase, step and stride length and mean speed were shown to be significantly lower and the double support phase higher than controls; in addition, in subjects with moderate and severe ARWMC, cadence was lower than controls and in subjects with moderate ARWMC, step width was larger than mild ARWMC and controls. Step and stride length, and mean speed were significantly lower in moderate or severe than in mild ARWMC subjects, and stance and double support phases were longer. No gait parameter was significantly different in the severe compared to moderate ARWMC patients (Figure 1).

### 3.4. Correlation and Regression Model of the Gait with the Clinical-Radiological Data

The correlation coefficients of the normalised gait data and of the clinical-radiological parameters are reported in Table 2. The univariate analysis showed a high correlation coefficient (Table 2, r > 0.90) among stance, swing and double support, between stride time and cadence and between speed and swing speed; for this reason, we excluded further analyses of stance, swing, stride time and swing speed.

We observed that lower scores in mean walking speed and stride length and higher single and double support phases were associated with a low MMSE score; we further noticed lower scores of motor balance performance and functional level but no correlation with the mood status.

Finally, the decreases in motor performance and balance were correlated with the reduction of the functional and cognitive level but not with depression (Table 2 and Table 3). 

The multiple linear regression models are reported in Table 4 and Table 5.

In the linear regression model ARWMC, corrected for age, sex, weight and height, was significantly associated with stride length (R^2^ = 0.327), gait speed (R^2^ = 0.301) and double support time (R^2^ = 0.182). For these gait parameters, the models obtained with a forward stepwise selection of the clinical variables showed a significant increase in R^2^ values after the inclusion of NMPS and GDS (Table 3). Cadence and step width were not dependent on the ARWMC in our sample (Table 4).

The balance (BBS), functional (FIM) and cognitive (MMSE) scores were excluded from the model estimation because their partial correlations with the dependent variable were negligible (<−0.087) after the inclusion of the NMPS. The univariate association between these independent variables and the gait parameters was entirely explained by their association with the motor alterations expressed by the NMPS.

## 4. Discussion

We showed that the stride length, gait speed and double support time were significantly associated with the ARWMC severity; the motor performances (NMPS) explained a part of the relation between ARWMC and gait parameters and were associated with the gait parameters alteration stronger than the other independent variables.

In recent studies on healthy older adults with no history of strokes and not affected by dementia or Parkinson’s disease, the vascular lesions in the white matter and subclinical strokes were associated with slower gait speed, shorter stride length and longer double support time as we obtained [28,29]. A set of age-related vascular alterations probably contributes to the increased vulnerability of aged WM to hypoperfusion: WM receive blood mainly from the deep arterioles in the border zone between the middle and the anterior cerebral arteries; the arterioles in the aged WM can assume a tortuous course too, reducing the kinetic energy of the blood flow. There is also an excessive deposit of collagen in the venules, decreasing the lumen and the blood flow and impairing perivascular drainage [12]. Age-related WM changes may also occur as a result of free radical damage, degenerative changes in cells in the oligodendrocyte lineage and changes in microenvironments within WM [11,30].

The mood scores (GDS) have an effect on gait alteration, whereas the cognitive scores (MMSE) were not significantly related to gait data in our population of patients. More in detail, the models we created well represented this patient population, showing an almost strong multiple correlation coefficient summary values (R = 0.766), explaining up to 58,7% of the gait parameter variance. These findings have not been showed till now in the literature.

The gait parameters of patients with ARWMC differ from those of our healthy controls (Table 1) and adult subjects’ normative data [31,32] and are similar to those obtained in larger samples of patients in the literature [17,18,19]; but most of these studies missed the association of gait and MRI with other relevant clinical and functional alterations (motor performance, balance, mood, cognitive and functional level). Additionally, in longitudinal studies with a comprehensive analysis of confounding factors and covariates, instrumental analysis of gait parameters has not been used [33,34,35,36].

We confirmed that the subjects with mild ARWMC showed a small-stepped symmetrical gait with reduced walking speed and increased double-support times. In addition, in patients with moderate and severe ARWMC, we found a reduction in cadence and an increase in step width. Concerning gait alteration in relation to ARWMC, no gait parameter was identified that distinguishes and characterises subjects with moderate rather than severe ARWMC; so, gait alterations are detectable in mild subjects with ARWMC but tend to be similar in moderate respect to severe patients.

Studying all the gait parameters quantitatively at once, we had the chance to discover which gait data were associated with the motor or neuro-psychological dysfunctions: the alteration of stride length, gait speed and double support time were explained by the ARWMC severity, motor impairment and depressed mood state, whereas cadence and step width were not associated with the ARWMC severity. 

The gait and equilibrium have been assessed in the literature using advanced diagnostic software systems [37], using information obtained from wearable sensors [38], by smartphones and tablets, in order to detect the symptoms of central nervous system impairment, such as cognitive impairment and neurodegenerative diseases [39,40,41,42]. 

With advanced software analysis, the process of gait pattern alteration is short compared to the comprehensive clinical assessment. With specific regard to the computerised systems for basic spatial and temporal gait parameter analysis, many devices have been recently compared, including inertial sensors, pressure sensors and optical systems, with an overall high agreement and consistency between systems. An optical system similar to the one used in this study showed a higher agreement with pressure systems than inertial sensor systems [43]. The marker-based gait analysis, including the optical systems, such as the one used in this study, can simultaneously record joint kinematics and kinetics too. It can be used before and after treatment, including orthosis, rehabilitation or surgical approaches. A limitation of our system was the possible misplacement of surface markers and being a time-consuming method, requiring expensive technical equipment and trained staff. On the other side the markerless motion capture systems, such as floor sensors, with ease of handling, portability, low acquisition costs and cannot collect information on joint movement; active markerless systems require camera triangulation or Time-of-flight methods and depend on light conditions in a controlled environment lab [44]. Finally, markerless silhouette extraction-passive systems represent a novel system used mainly in tracking, estimation and recognition of human motion [45,46,47] and could be used in a natural external environment, with limitations of wearing different clothes, carrying bags or back-pack [48]. The identification of human gait pattern takes advantage of real-time video capture, which is also great for advanced video surveillance applications with very high accuracy [45,46]; many state-of-the-art semi-subjective and objective techniques for gait analysis have limitations that could be mitigated using advanced machine learning-based approaches [47], but not all movement analysis labs have access to this technology.

Latest generation wearable systems with miniaturised sensor systems have a growing application and a decreasing cost that makes it an enormous potential consumer product to be used outside of a laboratory setting in everyday life and more complex activities. Accelerometery-based devices called inertial sensors combined with gyroscopes and magnetometers are the most promising [49,50]; to date, these data have lower accuracy and cannot still be used in clinical and scientific settings; they have limited battery power and complex algorithms are needed to obtain parameters. The challenge will be to have wearable devices with high computing power, reliable algorithms and limited energy consumption. Systems that require less time and resources while still providing sufficient accuracy will be the future of gait analysis.

In order to improve the analysis of the gait pattern, studies on joint kinetics and kinematics can be integrated into spatial and temporal gait parameters taking advantage of the same technical equipment that we used. However, the more complex the analysis algorithm, the more a staff of biomedical engineers is needed for signal analysis. A step forward is therefore still needed in software development to be more easily used, universally accepted and clinically oriented.

In the multiple regression analysis, the patient’s stride length was further the gait parameter with overall best model summary values; it was associated with the ARWMC, corrected for age, sex, weight and height (R = 0.576, R^2^ = 0.327); the inclusion of the NMPS in the model, in addition to the corrected ARWMC, accounted for a 22% increase in the variance explained for the stride (*d*R^2^ *p* = 0.000), but also caused a sharp drop of the ARWMC partial correlations from the zero-order correlation and a decrease in the regression coefficient (Table 3). This means that part of the existing relation between ARWMC and stride length is dependent on the white matter changes within the motor system pathways. So, the motor alterations are certainly associated with MRI lesions and explain a part of the correlation with the gait changes, but the partial correlation of ARWMC severity with gait changes remains significant even after excluding the direct effect on the motor system; this means that the influence of MRI abnormalities on gait does not just depend on purely motor abnormalities.

According to the multiple regression models, gait alterations were also influenced by the presence of depression (disclosed in 67.5% of the patients); indeed, the GDS score is a significant predictor of gait parameter alterations but independent from ARWMC severity and motor performance. In fact, with the increasing severity of depression, double support times significantly increase, stride length and average speed decrease. The depression scores added a further 3.9% to the variance explained by the model, but slightly modified the partial correlations and the regression coefficients of ARWMC and NMPS, indicating its independent role in generating a gait alteration. Similar results were obtained by analysing the models for gait speed and double support time (Table 3).

A mutually reinforcing relationship has been shown between disability and depressive symptoms in late life [51]; transition to disability in subjects with ARWMC could lead to depressive symptoms [52], which in turn worsen functional ability. From the analysis of our data, it is reasonable to hypothesise that the patients were not depressed because of their reduced motor performances but that, as a consequence of depression, they may appear as patients with more severe gait abnormalities than they actually have. The factors influencing the presence of depression in older individuals with high disability are still poorly understood. Many vascular mechanisms affect the fronto-limbic and other pathways predisposing to depression; generalised cortical atrophy or focal medial temporal atrophy are also related to late-life depression and associated with cognitive impairment [53]; although we have not analysed the regional distribution of white matter changes in our old patients, we obtained similar data in our patients, such as late-life-depression in association to white matter changes and gait disturbances.

We observed a correlation between the deterioration of gait parameters and a reduction in the cognitive level. This result is in accordance with the findings of other authors [54,55,56], as motor and gait alterations precede and predict cognitive disorder itself, but in multiple regression analysis, this association was not statistically significant. This means that the cognitive status, explored by means of MMSE, did not add a significant explanation to the variance of gait parameters in the models.

### Limitations and Strengths

Our study has the following limitations. Similar to other teams who are studying gait, there are some general difficulties in geriatric populations: studying patients in lab and finding a balance between the need to highlight some results with a strong statistical power and the relevance of underlying this result in a ‘‘real-life’’ population [10].

First, our sample, recruited in a rehabilitation department, represents a symptomatic population with functional deficits and cannot be generalised to other populations. Given ageing-related sarcopenia, rehabilitation interventions were performed in our patients for basic strengthening, functional mobility, gait and balance training and self-care skills training [3,4,57]. Training protocols have a positive effect on both gait and balance, walking speed and strength and may allow greater autonomy and independence to carry out activities of daily living in an elderly population [58]. We did not explore the training effect on our patients being a cross-sectional study, but a longitudinal study will be mandatory in order to show this improvement. Gait alterations in these symptomatic patients were similar to those already shown in high-functioning older adults with quantitative gait analysis [18] or in neurological outpatients but with a non-quantitative analysis [33]. Further the study does not address why some people has a gait disorder and other has not it in the general population. Our results represent indeed a specific gait disturbance of patients who need a neurorehabilitation. 

Moreover, the use of MMSE to evaluate cognitive function lacks sensitivity for specific cognitive domains. This can explain our lack of a significant correlation between gait parameters and cognitive function. Future studies should address if a more specific cognitive assessment can identify such a correlation with gait patterns. 

Some studies explore the role of white matter degeneration in age-related sleep disruption [59]; white matter degeneration is intimately connected to frontal-subcortical tracts, particularly on the right side [60]. Poor sleep quality is associated with white matter degeneration in widespread frontal-subcortical tracts with right hemisphere preponderance [61]. Given that the frontal-subcortical tracts connect several sections that dictate sleep and wakefulness [62], disruption in such circuits presumably accelerates the development of sleep disturbances disrupting sleep spindles and slow waves. The deterioration of white matter fibre tracts can compromise the synchronous recruitment of the neurons in older sleepers to produce slow wave sleep [63]. This may be the underlying mechanism of how white matter degeneration causes sleep impairment: driving decreases in sleep oscillations [64] and impairing their cortico-thalamic propagation loops [65]. Chronic sleep deterioration may contribute to memory, attention and cognitive impairment. Unfortunately, we did not explore the sleep disturbances in our patients. Bells et al. 2019 showed a fundamental connection between white matter microstructure and neural synchronisation that may be critical for cognitive processing [66]; so, it would be desirable to analyse the consequences of white matter on sleep and cognition in future studies with more specific scale for sleep and cognitive domains.

Finally, we have not analysed the brain spatial localisation of the ARWMC: its impact on gait is likely to be a consequence of disruption of motor pathways; hence, it may be reasonable to hypothesise that the severity of ARWMC correlate with gait alteration impairment. On the other hand, the semi-quantitative radiological scoring method we adopted for ARWMC has been previously demonstrated to have good reliability and sensitivity in cross-sectional studies [67] and has been extensively applied [33,35,52,68] and, as such, we used it as a relative point of strength. 

In any case, we are aware that the cross-sectional design also precludes us from making causal inferences regarding the association of ARWMC and gait that we found, so a longitudinal approach will be desirable in subsequent studies in order to show improvement after treatment and categories of the patient that best benefit of training protocols.

## 5. Conclusions

Gait analysis is non-invasive, quantifiable, and permanent (results can be stored on a hard drive or printed out). We have described the gait alterations that characterise patients with different ARWMC severities and associated deterioration of motor performance. The gait disorders in patients with ARWMC are related to motor impairment, but the presence of depression is an independent factor for determining gait alterations and functional status. The reduction of independence level in these patients caused by the gait disorder needs a combined neuro-rehabilitative and pharmacological treatment, tailored to each patient according to gait alteration and mood disorder, in order to prevent a deterioration of gait performance and a decline of the functional status. Future studies could highlight the role of gait analysis as an evidence-based tool to quantitatively assess gait changes after treatment or to monitor the natural progression of the gait disorder.

## Figures and Tables

**Table 1:** Baseline demographic and anthropometric data, gait parameters and clinical scores in the subjects grouped by ARWMC severity.

Demographic and Anthropometric Data	Controls (n = 14)	Mild ARWMC (n = 15)	Moderate ARWMC (n = 25)	Severe ARWMC (n = 36)	Tot Pat (n = 76)	F	p (ANOVA)
Age (years)	75.8 ±5	76.6 ± 5.5	79 ± 6.1	78 ± 6.6	78.3 ± 6.2	1.1	0.324
Sex (males. females)	6M, 8F	9M, 6F	14M, 11F	25M, 11 F	48M, 28F	1.0	0.369
Weight (kg)	63.8 ± 5.8	74.9 ± 15.5 *	68.1 ± 14.4	69.3 ± 15.5	70 ± 15.1	1.5	0.208
Height (cm)	162.3 ± 7	164.8 ± 10.2	165 ± 11.2	163 ± 10.5	164.4 ± 10.6	0.2	0.876
Gait parameters							
Stance (%)	61.7 ± 3.1	65.1 ± 4.9 *	70.1 ± 5.7 # °	72.6 ± 6.2 §	70.3 ± 6.3	16.1	<0.0001
Double Support (%)	23.1 ± 6.2	32.6 ± 8.5 *	40.7 ± 11.1 # °	45.0 ± 12.4 §	42.2 ± 12	15.8	<0.0001
Cadence (step/min)	106.2 ± 12.7	92.3 ± 18.3	79.3 ± 23.6 °	82 ± 20.1	83.1 ± 21.2	6.6	<0.0001
Swing speed (m/s)	2.27 ± 0.41	1.55 ± 0.38 *	0.95 ± 0.36 # °	0.93 ± 0.34 §	1.0 ± 0.4	54.0	<0.0001
Swing speed (%h/s)	140.2 ± 24.0	94.3 ± 22.3 *	57.8 ± 21.1 # °	57.1 ± 20.2 §	64.7 ± 25.4	60.7	<0.0001
Stride (m)	1.11 ± 0.11	0.8 ± 0.23 *	0.51 ± 0.21 # °	0.43 ± 0.18 §	0.53 ± 0.24	48.5	<0.0001
Stride (%h)	68.6 ± 6.5	48.8 ± 13.4 *	30.7 ± 12.1 # °	26.7 ± 10.7 §	32.4 ± 14.3	55.5	<0.0001
Step width (m)	0.16 ± 0.02	0.18 ± 0.03	0.21 ± 0.04 # °	0.20 ± 0.04	0.20 ± 0.04	6	0.001
Step width (%h)	10.1 ± 1.6	11.3 ± 2.1	12.9 ± 2.6 # °	12.6 ± 2.4	12.5 ± 2.5	5.4	0.002
Speed (m/s)	0.95 ± 0.18	0.58 ± 0.19 *	0.30 ± 0.16 # °	0.28 ± 0.13 §	0.35 ± 0.19	66.1	<0.0001
Speed (%h/s)	58.6 ± 10.6	35.6 ± 11.7 *	18.4 ± 9.7 # °	17.6 ± 8 §	21.4 ± 11.7	72.1	<0.0001
Clinical Scores							
MMSE (max = 30)	28.4 ± 1.3	26 ± 3.7	24.7 ± 4.2 °	21.4 ± 6.1 + §	23.4 ± 5.4	8.6	<0.0001
GDS (max = 30)	4.8 ± 2.1	14.4 ± 7 *	13.4 ± 7.7 °	14.6 ± 7.1	14.2 ± 7.2	7.6	<0.0001
FIM (max = 126)	125.6 ± 0.8	113.2 ± 17.9	102.5 ± 19 °	91.3 ± 27.4 + §	99.3 ± 24.5	9.8	<0.0001
FIM (^motor scores)^	90.8 ± 0.5	81.9 ± 13.6	73.7 ± 15.6 °	65.8 ± 20.7 §	74.5 ± 18.6	8.8	<0.0001
FIM ^(cognitive scores)^	34.8 ± 0.3	30.6 ± 5.2	28.8 ± 5.0 °	25.5 ± 8.1 + §	28.7 ± 6.9	8.0	<0.0001
NMPS (max = 14)	13.8 ± 0.5	9.6 ± 3.2 *	6.2 ± 3 # °	5.4 ± 3 §	6.5 ± 3.4	33.3	<0.0001
BBS (max = 56)	55.8 ± 0.5	45.4 ± 11.2 *	33.1 ± 14 # °	29.6 ± 16.2 §	33.9 ± 15.6	15.2	<0.0001

Abbreviations: ARWMC = Age Related White Matter Changes; MMSE = Mini Mental State Examination; GDS = Geriatric Depression Scale; FIM = Functional Independence Measure; NMPS = Nevitt Motor Performance Scale; BBS = Berg Balance Scale. Controls = Absent ARWMC; Mild, Moderate and Severe ARWMC= patients classified by means of Fazekas scale; results expressed as Mean ± SD (standard deviation); post hoc LSD analysis has been used for groups comparison: * *p* < 0.05 between absent and mild ARWMC group; # *p* < 0.05 between mild and moderate group; + *p* < 0.05 between moderate and severe group; ° *p* < 0.05 between controls and moderate group; § *p* < 0.05 between mild and severe group.

**Table 2:** Correlation coefficients of the normalised gait parameters.

Gait Parameters and Clinical Scores vs. Gait Parameters	Stance (%)	Swing (%)	Double Support (%)	Stride Time (s)	Cadence (Step/min)	Swing Speed (% h/s)	Stride (% h)	Step Width (% h)	Speed (% h/s)
Stance (%)	1	−0.957 **	0.954 **	0.530 **	−0.421 **	−0.652 **	−0.767 **	0.204	−0.767 **
Swing (%)	−0.957 **	1	−0.980 **	−0.593 **	0.492 **	0.643 **	0.684 **	−0.164	0.783 **
Double Support (%)	0.954 **	−0.980 **	1	0.580 **	−0.487 **	−0.694 **	−0.727 **	0.212	−0.761 **
Stride time (s)	0.530 **	−0.593 **	0.580 **	1	−0.907 **	−0.540 **	−0.245 *	0.07	−0.541 **
Cadence (step/min)	−0.421 **	0.492 **	−0.487 **	−0.907 **	1	0.554 **	0.189	0.023	0.539 **
Swing speed (% h/s)	−0.652 **	0.643 **	−0.694 **	−0.540 **	0.554 **	1	0.853 **	−0.256 *	0.956 **
Stride (% h)	−0.767 **	0.684 **	−0.727 **	−0.245 *	0.189	0.853 **	1	−0.298 **	0.886 **
Step width (% h)	0.204	−0.164	0.212	0.07	0.023	−0.256 *	−0.298 **	1	−0.288 *
Speed (% h/s)	−0.767 **	0.743 **	−0.783 **	−0.541 **	0.539 **	0.956 **	0.886 **	−0.288 *	1
ARWMC	0.428 **	−0.368 **	0.378 **	0.124	−0.144	−0.484 **	−0.540 **	0.158	−0.516 **
MMSE	−0.269 *	0.280 *	−0.299 *	−0.191	0.117	0.349 **	0.353 **	−0.189	0.323 **
GDS	0.384 **	−0.392 **	0.404 **	0.303 **	−0.273 *	−0.334 **	−0.317 **	0.091	−0.351 **
FIM	−0.350 **	0.345 **	−0.388 **	−0.201	0.124	0.452 **	0.481 **	−0.21	0.431 **
NMPS	−0.457 **	0.436 **	−0.495 **	−0.241 *	0.196	0.637 **	0.658 **	−0.321 **	0.631 **
BBS	−0.392 **	0.391 **	−0.445 **	−0.320 **	0.272 *	0.618 **	0.565 **	−0.291 *	0.592 **

Abbreviations: ARWMC = Age Related White Matter Changes; MMSE = Mini Mental State Examination; GDS = Geriatric Depression Scale; FIM = Functional Independence Measure; NMPS = Nevitt Motor Performance Scale; BBS = Berg Balance Scale. Pearson correlation coefficients *(r)* and two-tailored significance are reported (** *p* < 0.01; * *p* < 0.05).

**Table 3:** Correlation coefficients of the clinical-radiological scores.

Gait Parameters and Clinical Scores vs. Clinical Scores	ARWMC	MMSE	GDS	FIM	NMPS	BBS
Stance (%)	0.428 **	−0.269 *	0.384 **	−0.350 **	−0.457 **	−0.392 **
Swing (%)	−0.368 **	0.280 *	−0.392 **	0.345 **	0.436 **	0.391 **
Double Support (%)	0.378 **	−0.299 *	0.404 **	−0.388 **	−0.495 **	−0.445 **
Stride time (s)	0.124	−0.191	0.303 **	−0.201	−0.241 *	−0.320 **
Cadence (step/min)	−0.144	0.117	−0.273 *	0.124	0.196	0.272 *
Swing speed (% h/s)	−0.484 **	0.349 **	−0.334 **	0.452 **	0.637 **	0.618 **
Stride (% h)	−0.540 **	0.353 **	−0.317 **	0.481 **	0.658 **	0.565 **
Step width (% h)	0.158	−0.189	0.091	−0.21	−0.321 **	−0.291 *
Speed (% h/s)	−0.516 **	0.323 **	−0.351 **	0.431 **	0.631 **	0.592 **
ARWMC	1	−0.351 **	0.027	−0.348 **	−0.433 **	−0.358 **
MMSE	−0.351 **	1	−0.176	0.602 **	0.528 **	0.496 **
GDS	0.027	−0.176	1	−0.256 *	−0.177	−0.209
FIM	−0.348 **	0.602 **	−0.256 *	1	0.730 **	0.757 **
NMPS	−0.433 **	0.528 **	−0.177	0.730 **	1	0.881 **
BBS	−0.358 **	0.496 **	−0.209	0.757 **	0.881 **	1

Abbreviations: ARWMC = Age Related White Matter Changes; MMSE = Mini Mental State Examination; GDS = Geriatric Depression Scale; FIM = Functional Independence Measure; NMPS = Nevitt Motor Performance Scale; BBS = Berg Balance Scale. Pearson correlation coefficients (r) and two-tailored significance are reported (** *p* < 0.01; * *p* < 0.05).

**Table 4:** A multiple linear regression model was calculated for the gait parameters significantly associated with the ARWMC severity in the 76 patients.

Dependent Variables	Model Summary Values	R^2^ Change	Coefficients of the Independent Variables	Zero-Order Correlations	Partial Correlations
Stride (% h) Entry	R = 0.572 R^2^ = 0.327	dR^2^ = 0.327 *p* = 0.000	ARWMC = −9.28 (Age, Sex, Weight, Height *p* > 0.05)	−0.54	−0.51
Forward stepwise	R = 0.740 R^2^ = 0.547	dR^2^ = 0.220 *p* = 0.000	ARWMC = −5.31 NMPS = 2.21	−0.54 0.65	−0.35 0.57
	R = 0.766 R^2^ = 0.587	dR^2^ = 0.039 *p* = 0.013	ARWMC = −5.54 NMPS = 1.97 GDS = −0.42	−0.54 0.65 −0.31	−0.38 0.53 −0.29
Speed (%h/s) Entry	R = 0.549 R^2^ = 0.301	dR^2^ = 0.301 *p* = 0.000	Intercept = 66.9 ARWMC = −7.72 (Age, Sex, Weight, Height *p* > 0.05)	−0.51	−0.51
Forward stepwise	R = 0.729 R^2^ = 0.532	dR^2^ = 0.230 *p* = 0.000	Intercept = 52.6 ARWMC = −4.40 NMPS = 1.84	−0.51 0.63	−0.35 0.57
	R = 0.757 R^2^ = 0.573	dR^2^ = 0.041 *p* = 0.013	Intercept = 62.8 ARWMC = −4.59 NMPS = 1.64 GDS = −0.356	−0.51 0.63 −0.35	−0.38 0.53 −0.29
Double supp (%) Entry	R = 0.427 R^2^ = 0.182	dR^2^ = 0.182 *p* = 0.013	ARWMC = 6.4 (Age, Sex, Weight, Height *p* > 0.05)	0.39	0.39
Forward stepwise	R = 0.576 R^2^ = 0.332	dR^2^ = 0.149 *p* = 0.000	ARWMC = 3.5 NMPS = −1.5	0.39 −0.49	0.23 −0.42
	R = 0.649 R^2^ = 0.421	dR^2^ = 0.090 *p* = 0.002	ARWMC = 3.8 NMPS = −1.2 GDS = 0.55	0.39 −0.49 0.40	0.27 −0.36 0.36

Abbreviations: ARWMC = Age Related White Matter Changes; MMSE = Mini Mental State Examination; GDS = Geriatric Depression Scale; FIM = Functional Independence Measure; NMPS = Nevitt Motor Performance Scale; BBS = Berg Balance Scale. We reported the summary values of each regression model, the R-squared changes, the coefficients of the significant predictive variables, the zero-order correlations and partial correlations. First, we entered Age, Sex, Weight and Height with ARWMC; then we proceeded with a forward stepwise selection of the independent clinical variables., i.e., Stride (%h) = −5.54 ARWMC + 1.97 NMPS − 0.42 GDS.

**Table 5:** A multiple linear regression model was calculated for the gait parameters not associated with the ARWMC severity in the 76 patients.

Dependent Variables	Model Summary Values	R^2^ Change	Coefficients of the Independent Variables	Zero-Order Correlations	Partial Correlations
Cadence (Step/min) Entry	R = 0.339 R^2^ = 0.115	dR^2^ = 0.115 p = 0.120	Intercept = 134.7 ARWMC = −5.7 (Age, Sex, Weight, Height p > 0.05)	−0.14	−0.21
Forward stepwise	R = 0.421 R^2^ = 0.177	dR^2^ = 0.062 p = 0.026	Intercept = 124.7 ARWMC = −3.2 BBS = 0.3	−0.14 0.27	−0.11 0.26
Step width (%h) Entry	R = 0.332 R^2^ = 0.110	dR^2^ = 0.110 p = 0.137	Intercept = 25.6 ARWMC = 0.5 (Age, Sex, Weight, Height p > 0.05)	0.15	0.16
Forward stepwise	R = 0.430 R^2^ = 0.185	dR^2^ = 0.114 p = 0.014	Intercept = 27.4 ARWMC = 0.1 NMPS = −0.2	0.15 −0.32	0.03 −0.29

Abbreviations: ARWMC = Age Related White Matter Changes; MMSE = Mini Mental State Examination; GDS = Geriatric Depression Scale; FIM = Functional Independence Measure; NMPS = Nevitt Motor Performance Scale; BBS = Berg Balance Scale. We reported the summary values of each regression model, the R-squared changes, the coefficients of the predictive variables, the zero-order correlations and partial correlations. First, we entered Age, Sex, Weight and Height with ARWMC; then we proceeded with a forward stepwise selection of the independent clinical variables., i.e., Stride (%h) = −5.54 ARWMC + 1.97 NMPS − 0.42 GDS.

## Data Availability

The databases are owned by the contact author, but for privacy and ethical reasons they have not been published; upon justified request they can be sent by mail.
